# Exploring antibiotic stewardship interventions within a One Health context: a scoping review

**DOI:** 10.3389/fpubh.2025.1707695

**Published:** 2026-01-26

**Authors:** Léo Delpy, Chloe Clifford Astbury, Olivier Kambere Kavulikirwa, Mouhamadou Moustapha Sow, Sandrina Vandenput, Cécile Aenishaenslin, Arne Ruckert, Ria Benko, Mary Wiktorowicz, Tarra L. Penney, A. M. Viens, Marion Bordier

**Affiliations:** 1 ASTRE, University of Montpellier, CIRAD, INRAE, Montpellier, France; 2 National Laboratory for Livestock and Veterinary Research, Senegalese Institute of Research in Agriculture, Dakar, Senegal; 3 Center for Sociological and Economics Studies and Research (CLERSE), CNRS, University of Lille, Villeneuve-d'Ascq, France; 4 School of Global Health, York University, Toronto, ON, Canada; 5 Global Strategy Lab, York University, Toronto, ON, Canada; 6 Department of Social and Preventive Medicine, School of Public Health, University of Montreal, Montreal, QC, Canada; 7 Faculty of Veterinary Medicine, Catholic University of Graben, Butembo, Democratic Republic of Congo; 8 Department of Public Health and Social Medicine, Faculty of Health Sciences, Gaston Berger University, Saint Louis, Senegal; 9 Health Science Application and Research Laboratory, Gaston Berger University, Saint Louis, Senegal; 10 Health Sciences Library, University of Liege, Liege, Belgium; 11 Département de pathologie et microbiologie, Faculté de médecine vétérinaire, Université de Montréal, Saint-Hyacinthe, QC, Canada; 12 Groupe de recherche en épidémiologie des zoonoses et santé publique, Faculté de médecine vétérinaire, Université de Montréal, Saint-Hyacinthe, QC, Canada; 13 Centre de recherche en santé publique de l’Université de Montréal et CIUSSS du Centre-Sud-de-l’Île-de-Montréal, Université de Montréal, Montréal, QC, Canada; 14 Faculty of Pharmacy, Institute of Clinical Pharmacy, University of Szeged, Szeged, Hungary; 15 Albert Szent-Györgyi Health Centre, Institute of Clinical Pharmacy, University of Szeged, Szeged, Hungary; 16 Emergency Care Department, Albert Szent-Györgyi Health Centre, University of Szeged, Szeged, Hungary; 17 Dahdaleh Institute for Global Health Research, York University, Toronto, ON, Canada; 18 Global Food & Health Systems Research, School of Global Health, York University, Toronto, ON, Canada; 19 CIRAD, ASTRE, Dakar, Senegal

**Keywords:** interventions, legal instruments, stewardship, One Health, antibiotic use and resistance, antimicrobial use and resistance

## Abstract

Antibiotic resistance (ABR) presents a global threat to human health, animal health, and the environment. While ABR is a natural phenomenon, the overuse and misuse of antibiotics in human health, animal health, and food production is a major driver of ABR. As interactions between humans, animals, and the environment are central to the emergence and spread of ABR, adopting a One Health approach is essential to effectively address the issue. In this context, a large range of antibiotic stewardship interventions has been developed to optimize the use of antibiotics. However, we lack a comprehensive overview of the landscape of antibiotic stewardship interventions from a One Health perspective. To address this gap, we conducted a scoping review of existing policy interventions. The literature review used the Preferred Reporting Items for Systematic reviews and Meta-Analyses extension for Scoping Reviews guidelines. We systematically searched three major databases to retrieve interventions meeting our inclusion criteria. We purposively sampled some of these interventions to illustrate the diversity of existing interventions. These sampled interventions were then assessed according to 26 variables related to their general characteristics, development and implementation mode, scope, One Health and equity dimensions, and impacts. We conducted a descriptive analysis on the data extracted to summarize the characteristics of the antibiotic stewardship interventions. The 29 selected interventions focus on communication with stakeholders and the general public, access to antibiotics and their usage, and antibiotic disposal. Regarding their One Health aspects, the interventions varied in terms of collaboration across sectors and levels, engagement with the private sector, and equity considerations. Strong livestock industry engagement, efficient legal and institutional frameworks and provision of alternatives to antibiotics acted as facilitators to intervention implementation and success. Interventions demonstrated effects in terms of changes in antibiotic use, antibiotic resistance, awareness, and practices, as well as impacts on animal health and productivity. This study highlights the persistent dominance of human health objectives in the design and implementation of One Health antibiotic stewardship interventions and the continuous need to strengthen evaluation of such interventions to better understand facilitators and barriers to their implementation as well as intervention impacts.

## 1 Introduction

Antimicrobial resistance (AMR) presents a global threat to human health, animal health, and the environment. According to O’Neill (1), AMR is projected to cause 10 million deaths annually by 2050. In this global crisis, antibiotic resistance (ABR) plays a critical role and was estimated to be responsible for 4.95 million deaths in 2019 (2). ABR is a complex phenomenon, posing a challenge shared across human, animal and ecosystem health. Resistance to bacteria spreads between reservoirs (human, animal, environment), and scales (from local to global) through the direct transfer of resistant bacteria but also through the exchange of resistant genes among bacteria (3, 4). Transfer of ABR between animals and humans can occur following direct contact between humans and livestock and companion animals carrying resistant bacteria, or through the food chain (5). Ingestion of food of animal or plant origin containing antibiotic residues may also promote the development of resistance. Soil and water serve as reservoirs for resistant bacteria and antibiotic residues, whose presence mainly results from contamination by human activities, such as hospital waste, agricultural practices, and pharmaceutical industry discharge (6). The spread of ABR is further exacerbated at the global scale through international travel and trade. While ABR is a natural phenomenon, the overuse and misuse of antibiotics in human health, animal health, and food production over the past decades has become a major driver of ABR, leading to increasing levels of resistance (3, 4). As interactions between humans, animals, and the environment are central to the emergence and spread of ABR, and because the same classes of antibiotics are used in these different sectors, adopting a One Health approach is essential to effectively address the issue (7).

In this context, international organizations, scientific communities, and governmental authorities are advocating for the implementation of antibiotic stewardship (ABS) interventions to optimize antibiotic use (ABU), with the aim to curtail the ABR issue. ABS refers to using antibiotics appropriately to conserve their efficacy while ensuring their accessibility for those in need. However, there is currently no universal definition of ABS interventions, especially within a One Health perspective. Hibbard et al. (8) proposed a harmonized definition of ABS applicable to both human and animal health sectors, aiming to facilitate intersectoral communication and collaboration. However, this definition does not provide a clear benchmark for determining whether an intervention qualifies as One Health. This has led to a proliferation of definitions, varying in scope and scale depending on the implementation settings, the perspectives of stakeholders initiating the intervention, and the targeted audience.

Previous scoping reviews of antibiotic stewardship interventions have been undertaken, but have been focused on a specific sector, mainly in healthcare settings or in livestock production. We conducted a scoping review to explore the landscape of ABS interventions from a One Health perspective. The study offers a threefold contribution. First, it proposes a framework to define and characterize an ABS intervention within the One Health paradigm. Second, it identifies, characterizes and categorizes existing types of One Health ABS interventions, detailing their scope, objective, implementation context, impact, and One Health dimensions, while providing concrete examples to illustrate this typology. Finally, the study highlights the key enablers and barriers regarding the effective implementation of One Health ABS interventions.

## 2 Materials and methods

For this study, we performed a systematic search strategy to identify One Health ABS interventions, followed by a descriptive analysis of a selected sample of these interventions. The sample was purposively chosen from the identified documents to maximize diversity. This follows an emerging approach taken in existing qualitative evidence syntheses, which focuses analysis on a sub-set of identified documents to allow for an in-depth understanding of phenomena in context, in contrast with quantitative, effectiveness-focused syntheses which aim for exhaustiveness and statistical generalisability (9).

The scoping review was reported according to the Preferred Reporting Items for Systematic reviews and Meta-Analyses extension for Scoping Reviews (PRISMA-ScR) guidelines (10, 11) (see Supplementary File 1 for the PRISMA checklist).

### 2.1 Definitions and scope

We built on existing definitions of ABS interventions (12, 13) and One Health (14) to develop a definition of One Health ABS interventions for the purposes of this article. In order to keep the study manageable and because a scoping review about One Health surveillance of ABR has already been published (15), we took the decision to restrict the scope of this study to direct ABS interventions only (12). Consequently, in this study, we defined a One Health ABS intervention as an intervention designed to reduce ABU, control ABU, and/or promote appropriate ABU in order to preserve their current and future effectiveness, and which is implemented either: (i) in one sector (human, animal or environmental) with the aim to have positive effects in one or several other sectors; (ii) in one sector (human, animal or environmental) with the aim to have positive effects in this sector, with strong evidence that it has been developed based on integrated knowledge and information (e.g., generated by One Health surveillance systems); or (iii) in more than one sector (human, animal or environmental), with expected effects in one or several sectors.

### 2.2 Literature sources and search strategy

The literature search focused on peer-reviewed publications published between 1 January 2000 and 1 June 2024 in English, French, or Spanish. Based on the definition of a One Health ABS intervention adopted for this study, we identified four concepts to be characterized: “legal instrument”; “stewardship intervention”; “antibiotic resistance”; “antimicrobial resistance”; and “One Health.” An iterative approach was employed in collaboration with a librarian (SV) to select relevant keywords for each concept, guided by definitions provided in the Medical Subject Headings (MeSH) database. Search strategies were then developed for three databases: CAB Abstracts (EBSCO interface, 1973 onwards), Embase (Elsevier interface, 1974 onwards), and Medline (PubMed interface, 1946 onwards). See Supplementary File 2 for the complete and replicable algorithm.

### 2.3 Study selection

All documents retrieved from the bibliographic databases were uploaded to the Covidence software (Veritas Health Information, Melbourne, Australia), where duplicates were removed. The selection process was conducted in two steps by two independent reviewers (from among LD, OKK, and MMS). Disagreement between reviewers were resolved through discussion, and if necessary, a third author (MB or CCA) was consulted until consensus was reached.

In the first step, titles and abstracts were screened against four inclusion criteria: (i) the document describes a One Health ABS intervention that aligns with the study definition; (ii) the intervention(s) described in the document are formalized in an instrument issued by a recognized institution (e.g., international or regional organizations, governmental authorities, or professional organizations); (iii) the intervention(s) described in the document target antibiotics; and (iv) the document provides evidence that the intervention(s) described have been implemented, at least partially.

In the second step, all articles selected in the first step underwent a full-text assessment. An additional inclusion criterion was applied: the document must provide a detailed description of one or more One Health ABS interventions.

To ensure comprehensiveness, the bibliographies of selected publications were reviewed to identify additional relevant references, including scientific articles, national reports, guidelines, and regulatory documents.

Records that met all inclusion criteria were registered.

### 2.4 Data charting process

Building on previous work about interventions targeting ABU (12, 16), a data charting form was developed and iteratively refined during the data extraction process by a multidisciplinary team. This form was completed for each selected study by two independent authors (from among LD, MMS, CCA, and MB). The data extracted characterize the interventions described in the retrieved documents against a large set of variables (93), classified into seven categories: (i) general characteristics of the intervention (binding level, category, geographical area); (ii) development and implementation mode of the intervention (development process, organization involved in the development of the intervention); (iii) scope of the intervention (targeted bacteria, antimicrobials, reservoirs, transmission route and stakeholders); (iv) the One Health dimensions of the intervention (collaboration and equity); (v) the impacts of the intervention; (vi) the size of the intervention (number of beneficiaries, cost); and (vii) the socioeconomic context of the intervention (ABR level in the country, health care system, animal production system, legal system). Variables and their respective definition are available in Supplementary File 3.

As the study aimed to illustrate the diversity of existing interventions, we purposefully sampled some interventions in the initial database (9). This sampling approach, commonly used in evidence synthesis, supports detailed descriptions of each case (including example of interventions) and facilitates the development of a holistic understanding of a phenomenon (in this case, stewardship efforts to combat ABR from a One Health perspective). First, we identified variables for which sufficient information was available for the retrieved interventions and that captured the maximum variation across the interventions. At this stage we also collapsed some of the variables. We then selected interventions that differed from one another as much as possible with regard to these variables. For instance, we retrieved many similar national interventions that are translations of international guidance (e.g., national awareness campaigns implemented in the framework of the World Antimicrobials Awareness Week led by the WHO) or of regional regulations (e.g., national regulations adopted by EU countries to comply with their obligations in relation to the ban of antibiotics as growth promoters for food production), for which we only included one of each type. Once the interventions to be analyzed in depth were selected, additional searches were carried out through citation searching and reviews of coordinating institutions’ websites.

Additionally, we subsequently enriched the database with information about the context of the intervention implementation (i.e., governance system, legal system), drawing on World Bank country classifications (17) and supplementary internet research.

The final list of the 26 variables used to develop the database is presented in Table 1.

**Table 1:** Variables and their corresponding possible values used for the characterization of the One Health antibiotic stewardship interventions.

Variable name	Definition	Possible values
Intervention name	Name of the stewardship intervention	Name
Category	Category of the stewardship intervention	Information (interventions that improve knowledge and awareness about use of antibiotics), Access and use (interventions that regulate access and use of quality antibiotics), Disposal (interventions that regulate the elimination of antibiotics)
Sub-category	Sub-category of the stewardship intervention	Information: Community education, Consumer awareness, Public awareness, School-based programs, Decision support tools for prescription, Education of professionals, Training of professionals, Pledges; Access and use: Access restriction, Alternatives to antibiotics, Development of new antibiotics, Prohibition of use, Use restriction, Fight against substandard and falsified antibiotics, Prescription access only, Promotion restriction, Regulation of marketing, Regulation of sales and imports; Disposal: Safe disposal of unused or expired antibiotics, Reduction/prohibition of effluents from antibiotics manufacturing;
Document	Nature of the document where the intervention is described	Declaration, Policy, Regulation, Recommendation, Standard, Guidelines, Code of conduct
Year	Year of issue of the document describing the intervention	Year
Type of regulation	Type of regulation, when the document is a regulation	Public regulation (binding obligations in legal instrument), Self-regulation (non-regulatory binding measures but mandatory to meet professional obligation)
Binding level	Binding level of the intervention	Hard (legally binding obligations), Soft (non-binding measures that coordinate, guide, or shape individuals’ behaviors and practices)
Compliance source	Source of compliance of the intervention	Legal system, Industry standards, Professional codes and standards
Geographical scale	Scale of the territory where the intervention is implemented	International, Regional, National, Sub-national (at an administrative level: state, province, etc.)
Trigger	Reason for which the intervention has been developed	Preventive (the intervention is set up to anticipate a problem that has not manifested yet but is expected to happen), Reactive (the intervention is to cope with something that has already happened)
Purpose	Expected direct effects of the intervention	Residue reduction in food, Residue reduction in environment, Consumption reduction in humans, Consumption reduction in food animals, Consumption reduction in companion animals, Better prescription/usage practices, Preservation of last-resort antibiotics, Antibiotic resistance reduction
Antibiotic target	Type of drugs that the intervention targets	Antibiotics in general, Antimicrobials in general, Specific classes of antibiotics, Specific classes of antimicrobials, Specific antibiotics
Justification	The justification behind the development of the intervention	Compliance with international policies, Compliance with regional policies, Public health concern about ABR spread from animal to human, Public health concern about ABR spread from environment to human, Public health concern about preserving efficacy of last-resort antibiotics for humans, Public health concern about AB residues in food, Concern about the ABR rise in the animal sector, Concern about the ABR rise in human, Societal concern regarding healthy food, Societal concern about environment, Societal concern regarding animal health and welfare, Access to markets, Reputational reason
Leader	Sector(s) which has led the development of the intervention	Animal health, Environment, Human health, Food safety
Reservoir target	Main reservoir of antibiotic resistance or antibiotic residues that the intervention targets	Animal, Environment, Human, Plant
Transmission target	Main sequence of transmission that the intervention targets	Animal to animal, Animal to environment, Animal to human, Human to animal, Human to environment, Human to human, Environment to animal, Environment to environment, Environment to human, Plant to animal, Plant to human, Plant to environment, Plant to plant, All routes
Intervention implementer	Stakeholders in charge of implementing the measures prescribed by the intervention	Governmental authorities, Citizens in general, Patients, Farmers, Pet owners, Medical doctors, Veterinarians, Pharmacists, Other healthcare professionals, Feed producers and retailers, Food producer and retailer, Governmental authority, Pharmaceutical companies, Waste and wastewater management company
Effects observed	Final outcomes (both expected and unexpected) the intervention contributed to and the method used to measure them	Free text
Type of One Health intervention	Type of One Health intervention	Positive effect intervention (intervention implemented in one sector which is expected to have positive effects in one or several other sectors), Integrated information (intervention implemented in one sector with strong evidence that it has been developed based on integrated knowledge and information), Multisectoral intervention (intervention that has been implemented in more than one sector with expected effects in one or several sectors)
One Health collaboration	One Health collaboration during the development of the intervention	Multi-level (integration of the perspectives of people at the different levels of the administration and/or the society in the development of the intervention, especially the ones who will be impacted), Multi-sectoral (intervention has been developed within an intersectoral framework), Public-private partnership (collaboration between the public and private sectors in the development of the intervention)
Equity	Equity dimension taken into consideration in the intervention	Economic impact (effort to consider the economic impact of the intervention), Geographical (effort to deploy the intervention all over the territory intended to be covered by the intervention), Health (effort to balance the positive and negative health impacts of the intervention between sectors and populations), Language (effort in translating the intervention into a language that can be understood by all categories of targeted stakeholders), Socio-demographic (effort to take into consideration gender and all population categories, including the most vulnerable, in the development and implementation of intervention), No (existing evidence against equity consideration)
Barriers	Barriers to the success of the intervention	Free text
Enablers	Enablers to the success of the intervention	Free text
Legal system	Legal system in place in the country of implementation	Civil law, Common law, Mixed
Governance model	Governance model of the country of implementation	Centralized, Decentralized
Development level	Level of development of the country as per the World Bank classification	High-income countries, upper-middle-income country, low-and middle-income countries

Finally, we conducted a descriptive analysis of the compiled database to summarize the key characteristics of the selected One Heath ABS interventions. For free-text variables (namely justification, effects observed, barriers and enablers), qualitative content analysis was applied to available data to identify recurrent themes across the interventions.

## 3 Results

**Figure 1:**
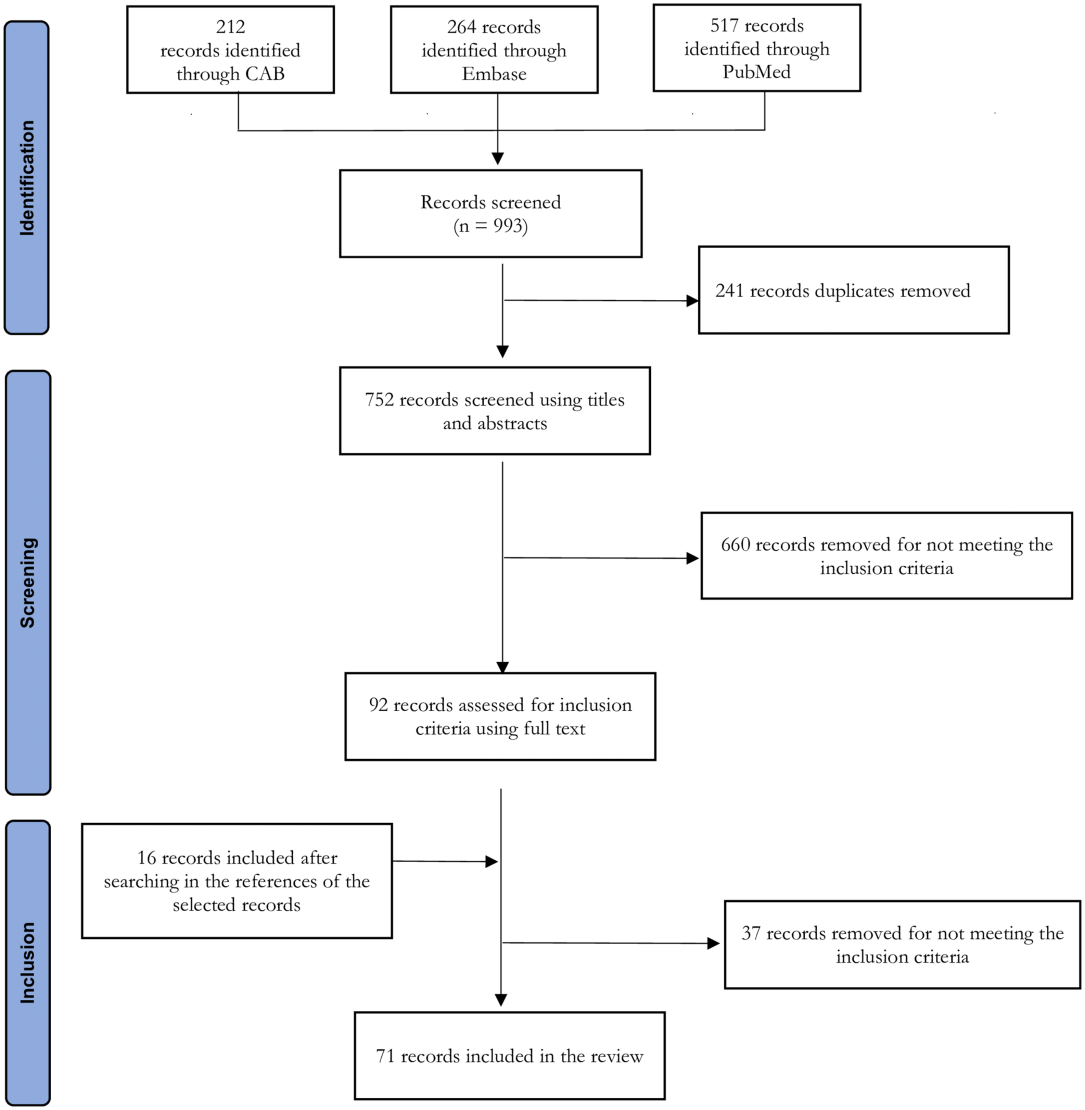
Preferred Reporting Items for Systematic reviews and Meta-Analyses extension for scoping reviews flow chart describing the study selection process for the scoping review.

The literature search identified a total of 993 records. After the screening process, 55 references were retained and 16 additional references were identified through reference searches of selected documents (Figure 1). From these 71 documents, we retrieved 61 interventions meeting our definition of a One Health ABS intervention, as in some cases the same interventions were described in multiple publications. Using purposive sampling for maximum heterogeneity on these 61 interventions, we obtained 29 interventions that represent the diversity of the ABS intervention landscape from a One Health perspective (see Table 2).

**Table 2:** Characteristics of the interventions.

Intervention name	Category	Sub-category	Document	Year	Drug target	Reservoir target	Transmission target	Type of One Health intervention	Equity
World AMR Awareness Week (34)	Information	Public awareness Community education	Recommendations	2015	AM in general	All	All routes	Multisectoral intervention	Language Geographical
European antibiotic awareness day (35)	Information	Public awareness Community education	Recommendations	2008	AB in general	All	All routes	Multisectoral intervention	Language Geographical
United Kingdom: Antibiotic Guardian campaign (22, 23)	Information	Public awareness Community education	Policy	2021	AB in general	Animal Human Environment	Animal to human Human to animal Human to environment Human to human Environment to human	Multisectoral intervention	Geographical Socio-demographic
Spain: Awareness videos for prudent use of antibiotics (28, 29)	Information	Public awareness Community education	Policy	2019	AB in general	Animal Human	Animal to human Human to human	Multisectoral intervention	Language
Kenya: Information, education and communication initiative (30)	Information	Public awareness Community education	Policy	NA	AM in general	Animal Human Environment	Animal to environment Animal to human Human to animal Human to human Human to environment Environment to animal Environment to human	Multisectoral intervention	Socio-demographic
California, US: Reporting about AMU in food animal products sold in grocery stores (66)	Information	Consumer awareness	Public regulation	2017	AM in general	Animal	Animal to environment Animal to human	Positive effect intervention	No
Europe: Ban of antibiotics as growth promoters (20, 21)	Access/Use	Prohibition of use	Public regulation	2006	Specific classes of AB	Animal	Animal to human	Positive effect intervention	Economic Health
Denmark: Ban of antibiotics as growth promoters (67–69)	Access/Use	Prohibition of use	Public regulation	1998	Specific classes of AB	Animal	Animal to human	Positive effect intervention	Economic Health
Sweden: Ban of antibiotics as growth promoters (20, 68, 70)	Access/Use	Prohibition of use	Public regulation	1986	Specific classes of AB	Animal	Animal to human	Positive effect intervention	Economic Health
Israel: Ban on the use of AM for growth promotion and metaphylaxis and ban on veterinary use of certain classes of AM critically important in human medicine (38)	Access/Use	Prohibition of use	Public regulation	2018	Specific classes of AM	Animal	Animal to human	Positive effect intervention	No
Thailand: Restriction of the use of antimicrobials in food animal production (31–33)	Access/Use	Prohibition of use	Public regulation	2015	Specific classes of AM	Animal	Animal to human	Positive effect intervention	Socio-demographic
China: Ban on the use of colistin as a feed additive in animals (44, 71)	Access/Use	Prohibition of use	Public regulation	2017	Specific antibiotics	Animal	Animal to human	Positive effect intervention	Health
Colombia: Ban on the use of colistin as a feed additive in animals (72)	Access/Use	Prohibition of use	Public regulation	2017	Specific antibiotics	Animal	Animal to human	Positive effect intervention	No
Canada: Voluntary cessation of use in poultry farming of AM critically important in human medicine (19, 25, 26, 41)	Access/Use	Prohibition of use	Self-regulation	2014	Specific classes of AM	Animal	Animal to human	Positive effect intervention	Health
WHO Guidelines on use of MIA in food-producing animals (18)	Access/Use	Use restriction	Guidelines	2017	Specific classes of AM	Animal	Animal to human	Positive effect intervention	No
Quebec (Canada): Restriction on the use in food animals of AM of very high importance for human medicine (36, 45, 48, 73)	Access/Use	Use restriction	Public regulation	2019	Specific classes of AM	Animal	Animal to human	Positive effect intervention	Economic
United States: Ban on the use of medically important AM for food-animal growth promotion (24)	Access/Use	Use restriction	Public regulation	2017	Specific classes of AM	Animal	Animal to human	Positive effect intervention	Health
California, US: Restriction of the use of antimicrobials in food-animal production (24, 74)	Access/Use	Use restriction	Public regulation	2018	Specific classes of AM	Animal	Animal to human	Positive effect intervention	N. A.
Denmark: Yellow Card initiative (37, 39)	Access/Use	Use restriction	Public regulation	2011	AB in general	Animal	Animal to human	Positive effect intervention	Geographical Health
Denmark: Antimicrobial treatment guidelines for pigs (37, 39)	Access/Use	Use restriction	Guidelines	2016	AM in general	Animal	Animal to human	Positive effect intervention	Geographical Socio-demographic
Denmark: Voluntary ban on use of cephalosporins in pig production (37, 42)	Access/Use	Use restriction	Recommendation	2010	Specific classes of AB	Animal	Animal to human	Positive effect intervention	Health
Thailand: Certification of pork products raised without antibiotics (31)	Access/Use	Use restriction	Standard	2015	AB in general	Animal	Animal to human	Positive effect intervention	Health
Thailand: Reduction of inappropriate use of antimicrobials in food crop through the promotion of good agricultural practices (31)	Access/Use	Promotion restriction	Standard	2013	AM in general	Plant	Plant to human Plant to environment	Positive effect intervention	No
United States: Removal of label claims for growth promotion, feed efficiency and/or improved weight gain for MIAs (24)	Access/Use	Promotion restriction	Recommendation	2013	Specific classes of AM	Animal	Animal to human	Positive effect intervention	Health
Canada: Removal of label claims for growth promotion, feed efficiency and/or improved weight gain for MIAs (27)	Access/Use	Promotion restriction	Recommendations	2005	Specific classes of AM	Animal	Animal to human	Positive effect intervention	No
United Kingdom (Midlands): A month-long amnesty for better management of unused and expired antibiotics (22)	Disposal	Safe disposal of unused or expired AB	Policy	2021	AB in general	Human	Human to human Human to environment	Positive effect intervention	No
WHO: Points to Consider for Manufacturers and Inspectors: Environmental Aspects of Manufacturing for the Prevention of Antimicrobial Resistance (75)	Disposal	Reduction/prohibition of effluent from AB manufacturing	Recommendations	2020	AM in general	Environment	Environment to human	Positive effect intervention	No
AMR industry alliance standards to reduce the environmental impact of antibiotics production (76)	Disposal	Reduction/prohibition of effluents from AB manufacturing	Standard	2017	AB in general	Environment	Environment to human	Positive effect intervention	N. A.
India: Draft regulatory standards for the pharmaceutical industry (40)	Disposal	Reduction/prohibition of effluents from AB manufacturing	Standard	2020	AM in general	Environment	Environment to human	Positive effect intervention	No

AB, antibiotic; AM, antimicrobial; AMR, antimicrobial resistance; AMU, antimicrobial use; MIA, medically important antimicrobials; N.A., not available; No, existing evidence against equity consideration; US, United States; WHO, World Health Organization.

### 3.1 General characteristics of stewardship interventions

Of the sample of 29 interventions analyzed in depth, six focus on the information of stakeholders and the general public regarding ABU (five related to public awareness campaigns, five to community education, one to consumer awareness), 19 on antibiotic access and use (eight related to prohibition of use, eight to use restriction, and three to promotion restriction), four on disposal (including three that reduce or prohibit effluent from antibiotic manufacturing, and one on the safe disposal of unused or expired antibiotics).

ABS interventions were described in 12 hard legal instruments and 17 soft legal instruments, among which 13 were regulations, four policies, seven recommendations, two guidelines and three standards. Regarding regulations, 12 were public regulations (hard legal instruments), and one was self-regulation (soft). The legal instruments were intended to be applied at an international (4 interventions), regional (2), national (19), or sub-national (4) level. They were all issued by governmental authorities, with the exception of three that were issued by the food production industry (2) or pharmaceutical industry (1). At national level, these instruments were implemented in low- and middle-income countries (LMICs) (1), upper-middle-income countries (6) and in high-income countries (HICs) (16). Half of the ABS interventions (15) were described in documents issued after 2015, year of adoption of the Global Action Plan by the World Health Assembly. 

All interventions were developed to address specific risk factors related to ABR, namely consumption reduction (22). better prescription or usage practices (13), and antibiotic residue reduction in food (5) or in the natural environment (3). preservation of the last-resort antibiotics for human medicine (7). Their ultimate aims were varied and sometimes overlapping. Some interventions aimed to protect human health, being developed in response to concerns regarding the spread of ABR from animals to humans (9), the spread of ABR from environmental reservoirs to humans (2), the loss of efficacy of last-resort antibiotics for humans (4), and the rise of ABR in humans (2). Others focused on animal health, aiming to address the rise of ABR in livestock animals (2). Some policy interventions were oriented toward the country’s position in the global sphere, including aiming to support compliance with international or regional policies and regulations (8), enabling access to international markets (6), or protecting international reputation (2). Interventions were developed under the leadership of the human health (7), animal health (16), food safety (13), or environment (1) sectors. They are expected to be implemented mainly by farmers (17), pharmaceutical companies (5), healthcare professionals (medical doctors, nurses, veterinarians, etc.) (5), the community in general (3), under the supervision of international/regional organizations, governmental authorities or professional organizations.

They target antibiotics in general (7), antimicrobials in general (7), categories of antibiotic or antimicrobials of very high importance in human medicine (12), or some specific antibiotics, such as colistin (2) or cephalosporins (1), in the following reservoirs: the animal reservoir (22), the environmental reservoir (5), the human reservoir (5), and the plant reservoir (1). Two interventions target all reservoirs. They all aim at minimizing hazard transmission (antibiotic residue or resistant bacteria) to human, either from animal (22), environment (5), human (4), or plant (1).

### 3.2 One Health characteristics of the ABS interventions

Among the 29 interventions selected, 24 were considered One Health because they were sectoral interventions expected to have positive effects in other sectors. For instance, the ban of antibiotics as growth promoters in livestock in the European Union primarily aimed at preserving the efficacy of antibiotics to treat bacterial infections in humans (45, 59). The remaining five interventions were considered One Health because they were interventions implemented in more than one sector. For instance, the Antibiotic Guardian campaign in the United Kingdom aimed at raising awareness about the judicious use of antibiotics both for animals and humans (28, 66).

While we identified interventions based on integrated information, they were all falling in the previous two categories (meaning that any of them were sectoral intervention expected to have positive effects in other sectors or multi-sectoral interventions), with no interventions identified that met our third definition of a One Health ABS intervention (i.e., an intervention implemented in one sector (human, animal or environmental) with the aim to have positive effects in this sector). Collaboration and equity are at the heart of the One Health approach as defined by the One Health High Level Expert Panel (14), and are variously expressed in the selected interventions.

#### 3.2.1 Collaboration

The selected One Health ABS interventions demonstrated collaboration between sectors, across levels, and through public and private partnerships, for the development and implementation of interventions.

Interventions aimed at raising awareness of ABR (6) or at restricting the use of medically important antibiotics for humans in food-producing animals (8) were usually developed through a strong collaboration between the human health, animal health, and food safety sectors (11). For instance, the regulation banning the use of medically important antimicrobials for food-animal growth promotion in the United States involved the Department of Health and Human Services, and the Food and Drug Administration’s Center for Veterinary Medicine (24). Conversely, the international Guidelines on Use of Medically Important Antimicrobials in Food-Producing Animals issued by the WHO (18) was developed with little contribution from the Food and Agriculture Organization (FAO) and the World Organization of Animal Health (WOAH); such input as they did provide was through their participation as special members of the WHO Steering Group.

Other interventions are the product of a partnership between the public and private sectors. For instance, in Canada, the recommendation to remove label claims for growth promotion, feed efficiency and/or improved weight gain for medically important antibiotics was supported by the government to promote judicious use of antibiotics; the intervention, however, was industry-driven to bring Canadian packaging in line with US legislation for export purposes. Consequently, health authorities and industry, albeit driven by different motivations, have worked closely together to push this recommendation into application (19, 25, 26).

Finally, we found evidence of collaboration between different operating levels and with the community for the development of some interventions. In Canada, a federal country, Health Canada’s Veterinary Drugs Directorate actively engages with provincial and territorial authorities to promote the judicious use of medically important antimicrobial drugs in food animal production, notably through the removal of growth promotion and/or production claims of medically important antimicrobial drugs (27). In the United Kingdom, the intervention for better management of unused and expired antibiotics demonstrates collaboration between health authorities as well as with medical doctors and retail pharmacists (22).

#### 3.2.2 Equity

Equity seeks to address unfair disparities that arise from unequal access to health resources, knowledge, and intervention. This study highlighted the consideration of five dimensions of equity in the development and implementation of the selected One Health ABS interventions: language equity, geographical equity, sociodemographic equity, economic equity, and health equity (see Table 1).

Some interventions demonstrate significant efforts to ensure language equity by translating materials into languages accessible to all targeted stakeholders. This is particularly true for interventions targeting public awareness and community education. For instance, the awareness campaign for prudent use of antibiotics in Spain is translated into the country’s main different languages (e.g., Basque, Catalan, and Spanish) (28, 29). Conversely, in Kenya, the awareness campaign is conducted only in English and not in Kiswahili, the country’s second official language, a main barrier to the success of the intervention (30). Translation enables more people to be reached, although there is no guarantee that the message will be understood. This can depend on the level of general and health literacy, as well as other aspects of messaging effectiveness.

Sociodemographic equity requires efforts to take into consideration all population categories, including the most vulnerable, in the development and implementation of interventions. This is connected to language equity, but can go beyond this to include intersecting population characteristics such as gender, ethnicity or education. The awareness campaign for prudent ABU in the United Kingdom integrates testimonials and YouTube videos which feature representatives from the different socio-economic, gender and ethnic groups living in the country (22, 23). Population equity also refers to the fact that an inclusive and systemic approach has been adopted to impact all sub-groups within the targeted population. For instance, Thailand deployed a specific effort to ensure that the national strategy to reduce ABU spanned all the food-producing sectors, including livestock, aquaculture and crop production (31–33).

Geographical equity can also be an intrinsic dimension of One Health ABS interventions, to ensure that interventions reach all regions in the targeted territory. For instance, initiatives developed by international and regional organizations, such as the World AMR Awareness Week organized by the WHO (34) and the European Antibiotic Awareness Day organized by the European Union (35), support member countries in implementing programs promoting the appropriate use of antibiotics, tailored to their context.

One Health ABS interventions also have implications for economic equity across the stakeholders concerned. In Quebec (Canada), discussions around the restriction on the use of antimicrobials of very high importance for human medicine in food animals hinted at issues of fairness and economic disparity (36). Farmers in this Canadian province were less competitive compared with neighboring provinces where similar restrictions were not in place. Financial impacts of One Health ABS interventions have also been observed for farmers in Denmark, where the ban of antibiotics as growth promoters in pig production has led to a loss of approximately 1 euro per pig on average (37). In Israel, documents underlined that the ban of certain antibiotics in food animals did not come with sufficient financial support from the government to mitigate the economic burden of the measures on farmers, such as support for the additional investment needed to boost biosecurity in the absence of the banned antibiotics (38). However, examples exist of interventions that sought to limit the economic impact for the targeted population. In the Danish Yellow Card initiative, for example, a threshold for antibiotics in farm animals is set at twice the average use per head of livestock. The restrictions are only applied to herds with use intensity over that threshold, meaning that only a small number of users are impacted (37, 39).

Health impact is unquestionably a core equity dimension of any One Health intervention. However, balancing the positive and negative impacts of interventions on the health of the different populations remains poorly taken into consideration in the retrieved One Health ABS interventions. The majority of interventions aim solely to strengthen the protection of human health without necessarily considering the wellbeing and health of animals or consequences for the environment. The justification of interventions targeting the appropriate use of antibiotics in animals is predominantly to keep antibiotics effective for humans and/or to avoid the release of antibiotic residues or resistant bacteria in the food chain, rather than to avoid the animal health and welfare consequences of overuse. When an intervention aims at reducing environmental contamination, such as the good manufacturing practices for the pharmaceutical industry (40), the purpose is to prevent medicines from becoming ineffective, rather than to protect the environment and biodiversity. However, there are some counterexamples. In Thailand, the main purpose of the certification of pork products raised without antibiotics is to ensure animal welfare in the farming process, supporting cruelty-free agricultural practices that resonate with consumers concerned about their wellbeing (31). In the Yellow Card initiative in Denmark, limits on ABU are set iteratively using ABU data provided by the Danish system for surveillance of the veterinary use of drugs for production animals (VETSTAT) and taking into account the health and welfare of animals. This approach helps avoid under-treatment that could lead to deterioration in the health and wellbeing of animals (37, 39).

### 3.3 Barriers and facilitators regarding the success of ABS interventions

The analysis of the selected One Health ABS interventions highlighted some determinants that may help or hamper the measures in producing their desired effects.

#### 3.3.1 Industry engagement

Collaboration between private and public actors has been the key to success for some interventions. In Canada, the poultry sector, represented by the Chicken Farmers of Canada, voluntarily withdrew the preventive use of Category I antimicrobials (i.e., antimicrobials considered to be of the highest importance to humans and used as a “last resort” in human medicine), including ceftiofur, in May 2014. This decision was prompted by evidence from the Canadian surveillance system, which showed that ceftiofur withdrawal in Quebec led to decreased resistance to third-generation cephalosporins in *Salmonella* isolates from retail chicken and sick individuals, as well as in *Escherichia coli* isolates along the food chain (41). Therefore, the positive effect of the intervention in Quebec, evidenced by surveillance data, allowed the diffusion of the intervention from one province to the entire country. In Denmark, synergistic action between the private and the public sectors helped to ban the use of cephalosporins in pig production (37, 42). Cephalosporins are considered to present a very high risk of the development of extended-spectrum beta-lactamases-producing bacteria and their transmission to humans. This led the Danish Agriculture and Food Council, representing the Danish farming and food industry including companies, trade organizations, and farmers’ associations, to implement a voluntary ban on cephalosporins for use in pig production. Shortly after the implementation of this, the Danish government introduced the “Yellow Card” scheme that aims to reduce the overall use of antibiotics in livestock, including cephalosporins. It consists of an administrative warning given to farmers exceeding the threshold and implying the payment of a flat fee, additional monitoring, and potential unannounced inspection visits at the farmer’s expense. Consequently, both in Canada and Denmark, engagement of an industry organization representing the actors targeted by the implementation of the measures was key to establishing efficient synergies between public and private actions targeting ABR reduction. In contrast, where there is a lack of effective partnering with the private sector, the implementation of interventions may be hindered. In California, the state government issued an ordinance requiring large grocery chains to report antimicrobial information for meat products sold in their stores (43). However, while grocery chains have answered questions about producer policies around AMU, the overall rate of reporting of figures on AMU has remained poor because of the beef and pork sectors’ unwillingness to provide AMU information (43).

#### 3.3.2 Implementation quality of the ABS interventions

The success of interventions depends not only on their design but also on the quality of their implementation. The implementation of the interventions included in this study were hampered by a number of factors.

First, legal and institutional capacity to enforce binding interventions was a barrier. In Thailand, for instance, the regulation restricting ABU in food animal and plant production had a mitigated impact. Antibiotic consumption in animals decreased by 49 percent following implementation of the regulations, but mainly because it targeted large agricultural food-production and -processing industries that account for 70–80% of the market share. However, the prescribing and dispensing of antibiotics are still weakly regulated by law and poorly audited, and farmers can purchase most antibiotics over the counter in pharmacies (31–33). Conversely, in China, the strong governmental commitment to monitoring the ban of colistin as a feed additive in animals is recognized as a main contributor to the estimated avoidance of more than 8,000 tonnes of consumption per year (44).

Second, the lack of standards and tools to operationalize the measures can also present a challenge. For interventions to regulate effluents from pharmaceutical companies that produce antibiotics, the main barrier to implementation is the current lack of established standards, although norms such as no-effect concentrations or minimal selective concentrations have been developed for certain antibiotics in certain contexts (40). In theory, the lack of standards necessitates applying the precautionary principle, which requires avoiding any waste discharge until it can be demonstrated that the effluents do not pose a risk to human health or the environment (40). However, in practice, antibiotic residues continue to be discharged into the environment without clear understanding and measurement of their impacts. Additionally, this sector is still missing a standardized method to define appropriate sampling methods and conduct laboratory testing (40).

Finally, the absence of alternatives to ABU can be a barrier to implementation. The existence of alternatives to those antibiotics whose use is restricted by regulations is key for acceptability and implementation by the targeted stakeholders. For instance, in Thailand, where citrus producers do not have alternatives as effective as antibiotics to treat their trees, antibiotic treatments are still widely used despite the efforts of the government to curtail this practice (32). Similarly, in Quebec, the restriction on the use on animals of antibiotics of critical importance in human medicine has met with resistance among farmers as they lack access to alternatives to prevent and treat diseases (45). Conversely, in China, the success of the ban on colistin is attributed in part to the fact that farmers adopted new disease management practices, relying on antibiotics not used in human medicine and supplemented by traditional Chinese medicines (44). The same observation has been made in Denmark where the ban of cephalosporins in pig production was well accepted and applied because producers observed a limited impact of the ban on animal health and welfare as other therapeutic options were still available to them (39).

### 3.4 Effects of the ABS interventions

A range of effects of implementation of the interventions are described in the selected documents, though evaluation methods and robustness are variable. Effects are described on: antibiotic consumption; levels of ABR; awareness and practices; and animal health and productivity.

#### 3.4.1 Changes in antibiotic consumption

Most interventions aimed at restricting ABU have seen reported evidence of reduced antibiotic consumption. However, it is often difficult to determine the specific contribution of a single intervention, as multiple initiatives typically work synergistically to reduce ABU within a given geographical area. Evidencing this ABU reduction is possible for countries where robust surveillance systems are in place to monitor antibiotic sales and consumption. For instance, in Canada and Denmark, authorities have been able to quantitatively measure the effect of their regulation to restrict ABU in livestock production since their respective national surveillance systems went into operation [2002 for the Canadian Integrated Program for Antimicrobial Resistance Surveillance (CIPARS) (41) and 1995 for the Danish Programme for Surveillance of Antimicrobial Consumption and Resistance (DANMAP) (46)]. In Denmark, the voluntary ban on use of cephalosporins in the pig production led to an overall reduction in therapeutic use of almost 25 percent after 2 years of implementation. In Quebec, a reduction in antibiotic consumption was observed following the implementation of regulations restricting the use of Category I antibiotics in food animals. In dairy herds, total sales of Category I antimicrobials, estimated in Canadian defined-course doses for cattle (DCDbovCA) fell from 14,258 to 21,528 DCDbovCA/month before the regulation to 1,494–4,707 DCDbovCA/month, without any increase in sales of other antimicrobials (36). Where robust surveillance systems are absent, impacts on ABU are challenging to assess. For example, in Israel, assessment of the effects of the ban on the use of antibiotics for growth promotion and metaphylaxis, and of the ban on veterinary use of antibiotics that are critically important in human medicine, was hampered by the lack of available data related to farm-level use of antimicrobials and antimicrobial sales by wholesalers, which was partly driven by the private sector’s desire to protect confidential commercial information (38).

#### 3.4.2 Changes in levels of antibiotic resistance

In some countries with well-advanced surveillance systems, as well as epidemiological and statistical capacities, the impact of ABS interventions on ABR levels are well described. Thanks to the well-resourced CIPARS network, Canada was able to observe that changes in ceftiofur use in broiler chickens were associated with variations in the proportion of ceftriaxone-resistant human and chicken isolates (25). Following the extension of the ban to other critically important antibiotics, surveillance results also demonstrated an overall decrease in use and resistance for most antibiotics in broiler chickens. However, although hatcheries in the neighboring province of Ontario had never announced an official withdrawal of ceftiofur use, a drop in ceftiofur resistance was also observed among chicken *Salmonella* Heidelberg isolates in Ontario in 2005 (19). Some argue that this indicates the absence of an association between ceftiofur use and ceftiofur resistance. However, the movement of hatching eggs, broiler chicks and retail chicken meat between Quebec and Ontario may explain the observed decrease of ceftiofur resistance in Ontario (25). In China, an epidemiological survey evidenced a decrease in the mcr-1 gene in pig farms following the ban of colistin as feed additive. However, authors of this study also acknowledged some potential biases in the results as the sampling intervals and the limited sample size could not capture seasonal effects (16).

#### 3.4.3 Changes in awareness and practices

Evidence of behavioral change following implementation of interventions to improve awareness, education and practices is less well documented, likely because such changes are difficult to assess. In the United Kingdom, actions to improve the management of unused and expired antibiotics in the community have been implemented. While some articles report changes in awareness, these changes are challenging to robustly substantiate. However, the interventions allowed for the collection of over 500 full or partial packs of antibiotics through collection points installed in 300 pharmacies across the Midlands in 2021 (22). At a global level, changes in practices have been observed in the antibiotic manufacturing sector, where an industry-led initiative has established standards and guidelines to control effluent emission through the calculation of the amount of active pharmaceutical ingredients discharge into the environment (40). Audits of companies complying with this initiative found that the manufacturing of 87 percent of products fell within the predicted no-effect concentration level, below which there are no foreseen adverse effects on the environment and the risk of promoting ABR is considered minimal. Some interventions have had some indirect effects on practice changes. For instance, following the implementation of the Yellow Card initiative in Denmark, which aims at reducing the consumption of antibiotics in pig production, an increase in vaccination against porcine circovirus type 2 (PCV2) related infections, gastrointestinal diseases, and respiratory infections was observed (39).

#### 3.4.4 Effects on animal health and productivity

Restricted use of certain classes of antibiotics in some populations may cause unintended harm to health and wellbeing, as well as a negative impact on productivity and producer incomes. In Europe, following the ban of antibiotics as growth promoters for animal production, Casewell et al. (20) describe an increase in morbidity and mortality in animals, as well as lower growth rates, with economic impacts for livestock owners (20). However, other studies emphasize the limited effects of ABU reduction on health and productivity. In Sweden and Denmark, similar bans were found to have no or limited impact on growth promotion in broilers and finisher pigs, even if some loss of growth in post-weaning pigs was reported, with some economic impact for producers (approximately 1 euro per pig in Denmark) (37). In the Netherlands, the reduction of the use of antimicrobials by approximatively 70 percent in the dairy sector from 2009 to 2015 did not affect animal health or welfare (47). Evaluating the isolated impact of an intervention on antibiotic reduction is difficult as producers usually implement alternative preventive measures that come at a cost (45).

## 4 Discussion

The study selected and described 29 interventions that enabled the characterization of the ABS environment from a One Health perspective. In response to the ABR health crisis, ABS is a growing field with broad applications, including residue reduction, consumption reduction, improved prescription and usage practices, and the preservation of last-resort antibiotics. These interventions are implemented across various sectors (human, animal, and environment), settings (healthcare facilities, animal and plant production systems, industries), and geographical scales (international, regional, national, and sub-national). From a One Health perspective, they vary in terms of inclusivity during their development and equity in their content. Although not always easy to assess robustly, most interventions achieved their main objective, namely to reduce ABU and/or decrease ABR, with some possible unexpected adverse effects on animal health and welfare and on the economy.

This study purposefully excluded from its search for ABS interventions any One Health institutional arrangements that may exist for ABR management at international level (such as the One Health High-Level Expert Panel), regional level (such as the Regional One Health Platform of the Economic Community of West African States) or national level (such as the national One Health platforms that are flourishing in many countries, especially in LMICs). However, we recognize that such governance mechanisms are key for the effective operationalization of the One Health concept applied to ABS, as long as there is strong ownership by and participation of stakeholders, their roles and missions are clearly defined relative to those of their members, and they have appropriate capacities for policy development (32).

### 4.1 The “One Healthness” of ABS interventions

In this study, we considered interventions to be One Health if they were implemented in one sector but with planned or expected impacts in another sector. However, the “One Healthness” of these interventions may be questioned when we note that almost all interventions with this cross-sectoral impact are designed with the objective of protecting human health. Interventions that restrict the use of antibiotics in the livestock sector are aimed at preserving their effectiveness for human treatment; of the ones selected for this study, almost none pay any consideration to the potential impact on animal health and wellbeing, beyond some economic considerations (with the exception of Thailand’s restriction of the use of antimicrobials in food-animal production, which cites animal welfare as an objective in its own right). One example of an intervention that clearly illustrates the anthropocentric tendency of current measures is the Guidelines on use of medically important antimicrobials in food-producing animals, issued by the WHO with little contribution from FAO and WOAH, the international organizations directly involved in animal health. The scientific community has questioned the impact of these anthropocentric measures on animal health and wellbeing (37, 48), pointing out that they have not been underpinned by initial risk-assessment studies to try to balance the negative and positive impacts between sectors as equitably as possible, and in particular to ensure that animals can still be treated while restricting antibiotic access to this sector. Accreditation and labeling that allow antibiotics when needed for therapeutic use, but emphasize reducing the need for these drugs by requiring minimum standards for husbandry and welfare, can help achieve this balance (49). In any event, the efforts to reduce antibiotic use fall mostly to the livestock sector, even though the sector makes a major contribution to food security internationally, and to livelihoods in regions of the world where livestock breeding is the main source of food and income for populations (50). The case of companion animals provides an interesting contrast as, despite the close interactions between companion animals and people and the high risk of transmission to human populations, we found limited examples of ABS intervention focused on companion animals. As the overall quantity of antibiotics used for this animal population is much lower than in livestock, ABR in companion animals is considered a lower priority by authorities and the scarcity of standardized data on ABU and ABR in pets limits the development of evidence-based interventions and hinders evaluation of their impact. In addition, owners’ relationships with pets are emotional and anthropomorphic, often leading to strong expectations for immediate treatment, and veterinarians may face pressure to prescribe antibiotics (51).

Additionally, this review did not retrieve interventions that were initially implemented with the primary or sole intention of protecting ecosystems. This might be due to the fact that the epidemiology and impacts of ABR circulating in ecosystems are largely unknown. When interventions are implemented in the human sector with the aim of protecting the environment (i.e., the control of effluent emissions from antibiotic manufacturing units), the final aim of such interventions is still to avoid further human exposure to antibiotic-resistant bacteria or residues from the environment. Nonetheless, some interventions evidenced an unplanned positive impact on the environment, as was the case for interventions aimed at recycling out-of-date or unused antibiotics, but without this being foreseen (22). Initially, these measures are implemented to prevent these antibiotics from being used in a way that is not optimal for human health. However, they also proved effective in preventing these antibiotics from being thrown away with household waste or emptied into household sinks, thereby contaminating the environment.

Thus, the prevailing conceptualization of ABS interventions is still anthropo-patho-centric: meaning that they focus not only on the human health aspects, but on the pathological elements of those aspects, giving little consideration to animal health and wellbeing or to the development of sustainable food systems, while undervaluing the wider human health or intrinsic benefits of preserved natural ecosystems. However, there is an emerging recognition that nature and animals are a greater source of health benefits than a reservoir of risks to humans, and also that animal wellbeing has inherent value in and of itself (52, 53).

This calls for a shift toward a more inclusive definition of One Health when developing ABS interventions to focus on health promotion rather than only on disease prevention and control, and on equity across sectors and populations (54). This implies a need to pay more attention to economic equity to ensure that interventions do not disproportionately burden the livestock sector, balancing costs with financial support mechanisms, access to affordable alternatives, and compensation schemes. Improving socio-demographic equity in ABS interventions would require tailoring awareness and training activities to different literacy levels, genders, and cultural contexts, and involving marginalized actors in the design and implementation of interventions. Health equity could be reinforced by systematically assessing both the benefits and unintended consequences of interventions on human, animal, and ecosystem health. Finally, geographical equity could be improved through the contextual adaptation of interventions to local governance capacities, infrastructure, and needs, especially in LMICs.

### 4.2 The need for evaluation

Developing effective and equitable ABS interventions from a One Health perspective requires evaluation of their outcomes and impacts to assess their balanced allocation across sectors. While many studies have shown that the use of antibiotics has led to increased occurrence of ABR (55), the body of scientific evidence on the effects of reducing their usage is far smaller. Interventions that include attempts to evaluate their effects are typically implemented in countries with operational integrated surveillance systems that produce high-quality data on ABR and ABU, as well as research institutions equipped to conduct complex data analysis. Still, even when those resources are available, it is difficult to attribute an observed effect to specific interventions, as the dynamics of ABR are very complex and often many interventions contribute to the same effect. For instance, in France, the ABR surveillance system has recorded that resistance to cephalosporins and quinolones in livestock has decreased since the regulation banning the use on livestock of critical antibiotics. But attributing the decrease to the regulation is challenging, as the regulation was part of a broader set of measures introduced in the national action plan (48). In Quebec, a decrease in ABU in dairy farms was observed following implementation of a new regulation restricting the use in livestock of antibiotics of very high importance for human health. However, at the same time the dairy industry implemented a program promoting wider biosecurity and traceability. In this context, it is challenging to determine the respective contributions of the regulation and of the adoption of new farming practices, which improved herd health, to the observed decrease of ABU (45). The challenges of evaluating interventions are further complicated by the multiple actors that implement them, including non-state actors. Industry self-regulation is a common aspect of ABS interventions, with a number of examples identified in this review, particularly in the livestock and pharmaceutical industries. While this industry leadership has driven progress in many contexts, evaluation remains critical to determine which interventions make a genuine contribution to health and which are more focused on self-positioning within the market, as the interests of commercial actors are not always aligned with health objectives (56).

Despite challenges in assessing the effectiveness of interventions and uncertainties in evaluation results, it remains essential to measure interventions’ direct and indirect impacts on productivity, as well as on animal health and wellbeing (45). Additionally, understanding the mechanisms of ABR gene dissemination is critical to refining restrictions and maximizing their effectiveness while ensuring fairness (48). This necessitates the development of specific attributes and indicators to evaluate ABS interventions from a One Health perspective (8). In addition, evaluation of One Health ABS interventions requires robust data not only on ABR and ABU but also on other determinants that contribute to ABR emergence and spread. This implies the need to extend the scope of surveillance beyond data on ABR and ABU to include factors such as climatic drivers (e.g., temperature and precipitation), meat consumption, and chemical levels that may contribute to the co-selection of resistance (15). Finally, it also seems necessary to strengthen knowledge of the mechanisms and processes that pave the change pathways toward the expected and unexpected outcomes of interventions and that may act as barriers to or levers for their effective implementation. Qualitative methods, such as realist evaluation and change-oriented evaluation approaches, could be used more systematically in the assessment of One Health ABS interventions (57, 58).

### 4.3 Policy diffusion in the antibiotic stewardship field

Our synthesis of One Health ABS interventions highlights the role of policy diffusion – a term referring to the influence of policies from one unit of governance on those of others (e.g., regions, countries, sub-national units)—on shaping policy globally (59). In this study, we found evidence of multiple mechanisms for policy diffusion at work. First, we identified instances of policy learning, where policy successes or failures in one unit inform decision-making around policy adoption in other units. This may be seen, for example, in the European Union, where a regional ban on antibiotics for growth promotion in livestock was implemented following previous bans in member states (Sweden and Denmark). In implementing this regulation, the European Commission referred to evidence of success in these countries, noting that by implementing these and other ABS policies, member states could substantially reduce their ABU with minimal impact on costs (20, 21). We also identified instances of policy competition, where units react to one another’s policy changes in an attempt to draw or preserve resources. Canada’s removal of claims of growth promotion and improved weight gain from the labels of medically important antimicrobials was an example of this. Veterinary drug manufacturers in Canada worked with the government to implement the policy and align with measures being taken by the US Food and Drug Administration. This step was taken by Canadian actors partially to strengthen North American trade and competitiveness (43). Finally, we identified instances of policy emulation, where a policy is adopted that is perceived as acceptable, expected, or beneficial to the reputation of the adopting unit. Thailand’s certification scheme for pork products raised without ABU, as part of a broader AMR national strategic plan, was an example of this. These policy changes strengthened Thailand’s reputation as an exporter of livestock and animal products and brought its regulations in line with international agency recommendations and regulatory environments in potential trading partners (33).

Policy diffusion is often influenced by structural and cultural similarities between jurisdictions, including legal systems, economic regulation, and language. For example, Quebec, as a French-speaking province and the only Canadian jurisdiction combining civil and common law, shares affinities with France and other French-speaking European countries, which tend to adopt more regulated market approaches and rely on binding legal instruments (i.e., hard law). In contrast, English-speaking provinces with common-law systems may align more closely with the United States, favoring a more market-driven approach that emphasizes persuasive legal instruments (i.e., soft law) or self-regulation by industry or professional organizations.

### 4.4 Learning but adapting

Our study provides a comprehensive overview of possible ABS interventions from a One Health perspective, together with their effects, as well as determinants influencing their effective implementation. It also found that an evolving global system of policy and governance exists, with countries learning from and reacting to each other as they attempt to curtail the global threat of ABR. However, ABS intervention efficacy is very dependent on the implementing context. An intervention that is successful in one setting might fail in another. The economic structure of a country, with its specific agricultural and livestock sectors, can significantly influence the nature of successful interventions (60). For instance, the success of the Yellow Card policy is directly linked to the structure of the Danish pig production system, where almost all producers and slaughterhouses are members of the same trade association and most producers operate with high biosecurity measures (39). The institutional and legislative context also greatly influences the implementation of interventions. An existing legal and institutional framework helps ensure the deployment of interventions, including the establishment of responsibilities, enforcement rules and accountability mechanisms. The adaptation of interventions to the governance structure and income level within a jurisdiction is crucial for effective implementation and compliance, especially in LMICs (61). Regulatory interventions on their own may be effective in countries with low corruption and high enforcement capacities. However, top-down, infrastructure-dependent interventions requiring strong regulatory oversight are less likely to be effective in curtailing ABR when used in isolation in LMICs. These approaches are more likely to succeed when combined with bottom-up ABR-containment strategies, which are often better accepted (62). Additionally, ABS interventions aimed at regulating access to antibiotics may be relevant to HICs where populations have access to well-equipped healthcare facilities and antibiotic misuse has been prevalent. But, for many LMICs, the health priorities lie in reducing morbidity and mortality due to bacterial infections, in a context of weak healthcare systems. International regulations and guidance, which promote global strategies shaped by HIC perspectives and concerns, may hamper the ability of LMICs to develop interventions tailored to their needs and constraints (15, 63). This is illustrated by the fact that the majority of LMICs have developed a national action plan to combat AMR with the support of international partners and in line with the WHO Global Action Plan, but that it is most often poorly operationalized due to a lack of ownership by national actors, insufficient domestic resources, and a lack of alignment with national priorities and local needs (64, 65).

### 4.5 Limitations

ABS interventions are mainly described in hard and soft legal instruments, which are not necessarily covered by the scientific literature or translated into English. Consequently, it is likely that our literature search missed some relevant interventions. National action plans are the most common soft law instruments described in the scientific literature. However, while these plans may include some ABS interventions that met the selection criteria of our study definition, many of the included actions may not yet have been implemented. As a result, we only integrated these interventions if we were able to find evidence of their actual implementation, or at least of efforts to apply them. Additionally, some interventions could not be selected because we could not retrieve enough information in the literature or our complementary searches to characterize them sufficiently. Additionally, the methodology adopted may have led to some limitations. In order to keep the number of articles to be screened manageable, the search strategy did not look into specific domains (e.g., animal species, environment reservoirs, human population) but at an integrated level across those domains as a whole (e.g., One Health, Planetary Health, etc.), and this has certainly excluded some articles from our selection In addition, we chose to focus our analysis on a sub-set of the interventions identified, selecting these interventions through purposive sampling for maximum heterogeneity. This means that some of the identified interventions were excluded from in-depth analysis, preventing a generalization of our findings, such as identifying the most common ABS interventions reported in the literature. However, this approach allowed us to meet our study objectives of illustrating the diversity of intervention mechanisms and models that exists in this space, describing the characteristics of our chosen cases in depth, and providing an overview of the wide variety of ABS interventions.

### 4.6 Conclusion

In analyzing the characteristics of 29 ABS interventions, this article provides a comprehensive examination of One Health approaches to reduce the use of antibiotics or promote their more appropriate use. Among the interventions analyzed, 24 were considered One Health due to their anticipated cross-sectoral benefits, mainly implemented in the livestock sectors with the aim of reducing antibiotic consumption in order to preserve the efficacy of antibiotics for human medicine or to avoid the presence of antibiotic residues in food of animal origin. The five remaining interventions were implemented across multiple sectors and were all related to communication and awareness about the necessity of prudent use of antibiotics.

This study offers several valuable contributions to the analysis of One Health ABS interventions. First, it introduces an analytical framework to describe such interventions. Second, it emphasizes the diversity and complexity of the One Health ABS landscape, providing some concrete examples of existing interventions, together with their respective objectives, some of the barriers to or facilitators for their effective implementation, and their success in reaching their expected results. Finally, it highlights the persistent dominance of human health objectives in the design and implementation of One Health ABS interventions, while noting the limited consideration for animal health and wellbeing, as well as for ecosystem health, in such initiatives.

The findings also stress the importance of tailoring interventions to the complexity and diversity of national and regional contexts. Future research should focus on how factors such as economic structures, institutional frameworks, and intersectoral collaborations influence the success of implementation of specific ABS interventions.
