# Supplementary material for: Exploring antibiotic stewardship interventions within a One Health context: a scoping review
Source: Front Public Health. 2026 Jan 26;13:1707695. doi: 10.3389/fpubh.2025.1707695 (PMC12883812; doi:10.3389/fpubh.2025.1707695)
Supplement: Supplementary file 2 [file Table_2.docx]

Supplementary file 2. Complete and replicable query - PubMed (June 20, 2023).

| Concepts | Search terms | Number of records |
| --- | --- | --- |
| Legal instrument | 'binding'/exp OR 'government'/exp OR 'prescribing guideline'/exp OR 'jurisprudence'/exp OR 'global health'/exp OR 'law'/exp OR 'management'/exp OR 'public policy'/exp OR binding OR binded OR campaign OR campaigns OR decree OR governability OR 'governance'/exp OR governance OR 'government'/exp OR government OR 'guideline'/exp OR guideline OR 'guidelines'/exp OR guidelines OR laws OR 'jurisprudence'/exp OR jurisprudence OR 'legal instruments' OR 'legal instrument' OR legal OR 'legislation'/exp OR legislation OR legislations OR legislative OR legislator OR legislating OR 'law'/exp OR law OR measure OR 'policy'/exp OR policy OR 'politic'/exp OR politic OR 'program'/exp OR program OR regulation OR regulatory | [7870959](https://www.embase.com/) |
| AND |  |  |
| Stewardship AND (antimicrobial resistance OR antibiotic resistance) | ('advertising'/exp OR advertising OR 'advertisement'/exp OR advertisement OR 'marketing'/exp OR marketing OR 'advertizing'/exp OR advertizing OR 'drug selling'/exp OR 'drug selling' OR 'authorization'/exp OR 'authorization' OR 'authorizations' OR 'authorisation'/exp OR authorisation OR 'awareness'/exp OR awareness OR ban OR bans OR 'coercion'/exp OR coercion OR 'consultation'/exp OR 'consultation' OR 'consultations' OR 'patient referral'/exp OR 'patient referral' OR 'referral and consultation'/exp OR 'referral and consultation' OR 'referral'/exp OR referral OR 'education'/exp OR 'education' OR 'educational' OR 'educative' OR 'educated' OR 'teaching'/exp OR 'teaching' OR 'educations' OR 'fee'/exp OR fee OR 'fees'/exp OR fees OR charges OR 'importation'/exp OR importation OR import OR incentivization OR incentivisation OR 'laboratory test'/exp OR 'laboratory test' OR 'laboratory tests' OR 'market'/exp OR market OR 'mitigation'/exp OR mitigation OR 'persuasion'/exp OR persuasion OR 'pharmacovigilance'/exp OR pharmacovigilance OR pharmacovigilances OR 'drug monitoring centre'/exp OR 'drug monitoring centre' OR 'drug monitoring center'/exp OR 'drug monitoring center' OR 'drug surveillance'/exp OR 'drug surveillance' OR pledge OR 'prescription'/exp OR prescription OR 'prescriptions'/exp OR prescriptions OR 'drug prescribing'/exp OR 'drug prescribing' OR 'audit'/exp OR 'audit' OR 'audits' OR 'diagnostic test'/exp OR 'diagnostic test' OR 'rapid testing'/exp OR 'rapid testing' OR 'rapid test'/exp OR 'rapid test' OR 'records'/exp OR records OR 'restricted reimbursement' OR 'restriction'/exp OR restriction OR sale OR 'shared decision making'/exp OR 'shared decision making' OR 'stewardship'/exp OR stewardship OR 'taxes'/exp OR taxes OR 'training'/exp OR training OR usage OR 'advertising'/exp OR 'clinical audit'/exp OR 'authorization'/exp OR 'awareness'/exp OR 'coercion'/exp OR 'patient referral'/exp OR 'health economics'/exp OR 'educational status'/exp OR 'teaching'/exp OR 'fee'/exp OR 'laboratory test'/exp OR 'marketing'/exp OR 'drug marketing'/exp OR 'mitigation'/exp OR 'pharmacovigilance'/exp OR 'prescription'/exp OR 'rapid test'/exp OR 'shared decision making'/exp OR 'tax'/exp OR 'education'/exp) AND  ('antibiotic resistance'/exp OR 'bacterial resistance' OR 'antibacterial resistance' OR 'antibiotic resistance' OR 'antibacterial drug resistance' OR 'microbial drug resistance' OR 'bacterial drug resistance' OR 'microbial resistance' OR 'antibiotic drug resistance' OR 'antimicrobial resistance' OR ABR OR 'antibiotic-resistant bacteria' OR 'drug resistance' OR ('chemotherapeutic agents' OR 'antimicrobial'/exp OR 'anti bacterial agents'/exp OR antimicrobial OR 'antibiotics'/exp OR antibiotics OR 'antibacterial'/exp OR antibacterial OR antimycobacterial OR 'anti mycobacterial') AND ('resistance'/exp OR resistance)) | 165951 |
| OR |  |  |
| 'antimicrobial stewardship'/exp) | 'antimicrobial stewardship'/exp | 10 111 |
| AND |  |  |
| Concept One Health | 'one health'/exp OR 'one health' OR 'multi stakeholder' OR 'multi stakeholders' OR 'one medicine' OR 'planetary health' | 17 311 |
| Total |  | 517 |
