# Supplementary material for: Exploring antibiotic stewardship interventions within a One Health context: a scoping review
Source: Front Public Health. 2026 Jan 26;13:1707695. doi: 10.3389/fpubh.2025.1707695 (PMC12883812; doi:10.3389/fpubh.2025.1707695)
Supplement: Supplementary file 3 [file Table_3.docx]

Supplementary file 3. Initial list of variables for the characterization of the antibiotic stewardship interventions from a One Health perspective.

| Variable group | Variable name | Definition |
| --- | --- | --- |
| Identification | Intervention name | The name of the intervention |
|  | Country of the intervention | Country where the stewardship intervention is intended to be implemented |
|  | Category | Category of the stewardship intervention |
|  | Sub-category | Sub-category of the stewardship intervention |
|  | Intervention nature | Short description of the measures of the intervention |
|  | Year | Year of issue of the document describing the intervention |
|  | Document | Nature of the document where the intervention is described |
|  | Type of regulation | Type of regulation, when the document is a regulation |
|  | Binding level | Binding level of the intervention |
|  | Compliance source | Source of compliance of the intervention |
|  | Geographical scale | Scale of the territory where the intervention is implemented |
|  | Overarching document type | Nature of overarching documents framing the intervention |
|  | Overarching document scope | Scale of the overarching documents framing the intervention |
|  | Purpose | Expected direct effects of the intervention |
|  | Reservoir target | Main reservoir(s) of ABR or antibiotic residues that the intervention targets |
|  | Transmission target | Main sequence(s) of transmission that the intervention targets |
| Development and implementation process | Development process | Description of the development process of the intervention (institutional arrangements, type of collaboration, actors involved and/or affected, etc.) |
|  | Trigger | Preventive or curative nature of the intervention |
|  | Organization number | Number of organization(s) (governmental, non-governmental, private) involved in the development of intervention |
|  | Organization name | Names of organization(s) involved in the development of intervention |
|  | Organization type | Type of organization(s) involved in the development of intervention |
|  | Organization sector | Sector of the organization(s) involved in the development of intervention |
|  | Inclusion | Level of inclusion of the actors impacted by the intervention in the development of the intervention |
|  | Leading organizations | Names of organization(s) leading the development of intervention (main leader group that initiated the intervention) |
|  | Leader type | Type of organization(s) leading the intervention development |
|  | Leader sector | Sector(s) which has led the development of the intervention |
|  | Supervisor name | Names of organization(s) in charge of supervising the implementation of the intervention |
|  | Supervisor type | Type of organization(s) supervising the implementation of the intervention |
|  | Supervisor sector | Sector of the organization(s) supervising the implementation of the intervention |
|  | Intervention enforcement mechanisms | Description of the enforcement mechanisms associated with the intervention |
|  | Financing organization name | Name of the organization(s) in charge of financing the implementation of the intervention |
|  | Financing type | Type of organization(s) in charge of financing the implementation of the intervention |
|  | Financing sector | Sector of organization(s) in charge of financing the implementation of the intervention |
| Scope | Categories of bacteria | The categories of bacteria that the intervention targets |
|  | Antibiotic type | Antibiotic classes or specific antibiotics that the intervention targets |
|  | Antimicrobials | Other antimicrobial categories than antibiotics concerned by the intervention |
|  | Implementing actor category | Category of the actor in charge of implementing the measures prescribed by the intervention |
|  | Implementing actor sector | Sector(s) of the implementing actor |
|  | Final target category | Category of the populations or commodities targeted by the expected outcomes of the intervention |
|  | Final target sector | Sector of the populations or commodities targeted by the expected outcomes of the intervention |
|  | Geographical name | The name of the territory where the intervention is implemented |
|  | Geographical scale | Scale of the territory where the intervention is implemented |
| One Health dimension | Type of One Health intervention | Type of One Health intervention (according to the study definition) |
|  | One Health dimension | Description of all elements that describe the One Health dimension taken into consideration during the development of the intervention |
|  | Multi-sectoral | Intervention developed within an intersectoral framework |
|  | Multi-disciplinary | Intervention developed using an interdisciplinary approach |
|  | Multi-level | Intervention took into account the perspectives of people at the different levels of the society |
|  | Barriers (One Health) | Barriers in integrating the One Health dimension in the intervention |
|  | Enablers (One Health) | Enablers in integrating the One Health dimension of the intervention |
| Impacts of the intervention | Effect observed | Demonstration of some effects following the implementation of the intervention |
|  | Effect direction | Direction of the observed effects of the intervention (positive, negative) |
|  | Effect type | Category of the effects of the intervention |
|  | Effect description | Description of the effects of the intervention |
|  | Effect expectation | Expected or unexpected nature of the effects observed |
|  | Evaluation | Conduction of an evaluation study to evidence the effects |
|  | Evaluation type | Type of the evaluation that have been conducted to evidence the effects |
|  | Evaluation description | Description of the evaluation that have been conducted |
|  | Barriers (effects) | Elements that have negatively impacted the implementation of the intervention and its effects |
|  | Enablers (effects) | Elements that have positively impacted the implementation of the intervention and its effects |
|  | Barriers (evaluation) | Elements that have negatively impacted the intervention evaluation |
|  | Enablers (evaluation) | Elements that have positively impacted the intervention evaluation |
|  | Negatively impacted stakeholder | Category of stakeholders who have been negatively impacted by the intervention |
|  | Negatively impacted population | Populations or commodities that have been negatively impacted by the intervention |
|  | Negatively impacted sector | Sector(s) of the impacted stakeholders and/or populations |
|  | Positively impacted stakeholder | Category of stakeholders who have been positively impacted by the intervention |
|  | Positively impacted population | Populations or commodities that have been positively impacted by the intervention |
|  | Positively impacted sector | Sector(s) of the impacted stakeholder and population belong to |
| Equity dimensions | Language equity | Existence of efforts in translating the intervention in a language that can be understood by all categories of targeted stakeholders |
|  | Language equity (description) | Free text |
|  | Geographical equity | Existence of efforts to deploy the intervention all over the territory intended to be covered by the intervention |
|  | Geographical equity (description) | Free text |
|  | Gender equity | Existence of efforts to take into consideration gender in the development and implementation of intervention |
|  | Gender equity (description) | Free text |
|  | Economic impact equity | Existence of efforts to take into consideration economic inequalities in the development and implementation of intervention |
|  | Economic impact equity (description) | Free text |
|  | Balancing intention | Existence of a clear desire to define an intervention that balances its benefits and burden between the sectors and populations (vulnerable, minority group, generations) |
|  | Balancing intention (description) | Free text |
| Costs | Intervention cost | The cost to develop and to implement the intervention |
|  | Intervention economic impact | The loss or gain observed due to the implementation of the intervention |
|  | Number of involved individuals | The number of individuals involved in the development, supervision, and implementation of the intervention |
|  | Number of beneficiaries | The number of individuals that are targeted by the intervention in the country of implementation of the intervention |
| Socioeconomic context | Health insurance coverage | Percentage of health insurance coverage in the country of implementation of the intervention |
|  | Animal Health expenditure | Public expenditure of animal health sector (% of total GDP) in relation to AMR in the country of implementation of the intervention |
|  | Human health expenditure | Public expenditure of human health sector (% of total GDP) in relation to AMR in the country of implementation of the intervention |
|  | Human Development Index | Human Development Index for the country of implementation of the intervention |
|  | Animal production | Quantity of animal production in the country of implementation of the intervention |
|  | ABR burden | Number of deaths related to ABR in the country of implementation of the intervention |
|  | ABR level | ABR levels of some key indicators in the country of implementation of the intervention |
|  | Law enforcement capacity | Capacity of the governmental authorities to enforce law in in the country of implementation of the intervention |
|  | Development level | Level of development of the country regarding the World Bank classification |
|  | Corruption | Level of corruption in the country of implementation of the intervention |
|  | Governance model | Governance model in the country of implementation of the intervention |
|  | Animal value | The value of domestic animals and wildlife in the society of the country of implementation of the intervention |
|  | Legal system | Legal system in place in the country of implementation of the intervention |

ABR: antibiotic resistance; AMR: antimicrobial resistance; GDP: gross domestic product
